# The Impact of Individualizing Sodium Bicarbonate Supplementation Strategies on World-Class Rowing Performance

**DOI:** 10.3389/fnut.2020.00138

**Published:** 2020-09-09

**Authors:** Susan Boegman, Trent Stellingwerff, Gregory Shaw, Nick Clarke, Kenneth Graham, Rebecca Cross, Jason C. Siegler

**Affiliations:** ^1^Canadian Sport Institute Pacific, Victoria, BC, Canada; ^2^Department of Exercise Science, Physical & Health Education, University of Victoria, Victoria, BC, Canada; ^3^Swimming Australia, High Performance Unit, Brisbane, QLD, Australia; ^4^Sport and Exercise Science Program, School of Health Sciences, Western Sydney University, Sydney, NSW, Australia

**Keywords:** sodium bicarbonate ingestion, individualized nutrition, time trial performance, elite athletes, performance

## Abstract

Contemporary meta-analyses have generally demonstrated a positive effect of sodium bicarbonate (NaHCO_3_) supplementation on exercise performance. However, despite these claims, there is limited data on contrasting individualized and standardized *timing* of NaHCO_3_ ingestion prior to exercise to further enhance performance outcomes.

**Purpose:** To determine whether NaHCO_3_ ingestion timing impacts 2,000-m rowing time-trial (TT) performance in elite-level rowers (Senior National team including Olympic/World Championships level) adhering to their own individualized pre-race strategies (e.g. nutrition, warm-up, etc.).

**Methods:** Twenty three (*n* = 23) rowers across two research centers (using the exact same methods/protocols) completed three trials: NaHCO_3_ loading profile at rest to determine the individual's time-to-peak bicarbonate concentration [${\text{HCO}}_{3}^{-}$], followed by two randomized 0.3 g·kgBM^−1^ NaHCO_3_ supplementation experimental trials conducted at different time points [consensus timing (CON): TT performed 60 min post-NaHCO_3_ ingestion; and individualized peak (IP): TT performed at the rower's individual peak [${\text{HCO}}_{3}^{-}$] determined from the profiling trial post-NaHCO_3_ ingestion].

**Results:** There was a significant mean difference of +2.9 [± 0.4 mmol·L^−1^ ${\text{HCO}}_{3}^{-}$ for IP vs. CON (95% CI 2.0 to 3.8 mmol·L^−1^); *p* = 0.02; *d* = 1.08] at pre warm-up, but not immediately prior to the TT (post warm-up). Performance times were significantly different between IP (367.0 ± 10.5 s) vs. CON (369.0 ± 10.3 s); *p* = 0.007; *d* = 0.15).

**Conclusions:** The present study demonstrated a small but significant performance effect of an individualized NaHCO_3_ ingestion strategy. Similarities after warm-up between pre-TT ${\text{sHCO}}_{3}^{-}$ values (CON ~ + 5.5 mmol·L^−1^; IP ~ + 6 mmol·L^−1^), however, would suggest this effect was not a result of any meaningful differences in blood alkalinity.

## Introduction

The most recent 2018 International Olympic Committee (IOC) sports nutrition consensus statement recommendations have suggested that sodium bicarbonate (NaHCO_3_) is one of five dietary supplements that has generally been shown to improve performance in the elite athlete (1). Indeed, there are a number of contemporary meta-analyses demonstrating the potential performance efficacy of supplementing with NaHCO_3_ as compared to placebo in sports where perturbations in cellular buffering capacity influences performance (typically in events of 1–10 min) (2–5). Current consensus statement ingestion recommendations are to consume between 0.2 and 0.4 g·kg body mass (BM)^−1^ with a small, carbohydrate (CHO) dense meal (~1.5 g·kgBM^−1^ CHO) ~60 to 150 min prior to exercise (1). However, as contemporary papers have also highlighted, these recommendations serve only as a starting point when considering NaHCO_3_ supplementation for the individual athlete (6–8). A recent review by Heibel et al. has addressed several practical issues associated with traditional NaHCO_3_ supplementation approaches, identifying ingestion timing as potentially critical to maximizing the effectiveness of this supplement (6). Given the existing scientific support for the use of NaHCO_3_ (2–5), as well as the high prevalence of use [e.g., rowing (9–12)], understanding the influence of ingestion timing under ecologically valid conditions may further improve the effectiveness of this supplement. To date no study has collectively investigated the relationship between NaHCO_3_ timing, buffering capacity, gastro-intestinal (GI) distress, and pre-race nutritional recommendations coupled to performance outcomes in world-class athletes.

The premise for individualizing NaHCO_3_ supplementation has both historical (8, 13) and more recent scientific support (14–16). Several contemporary publications have consistently demonstrated the high degree of inter- and intra-individual variability often observed during NaHCO_3_ studies, despite dosing by body mass (kg) and standardizing pre-supplementation nutrition and fluid intake (7, 8, 17). Furthermore, a relatively recent study has profiled the large individual variations in blood bicarbonate (${\text{HCO}}_{3}^{-}$) in response to ingesting 0.1, 0.2, and 0.3 g·kg^−1^ NaHCO_3_ (7). These authors also underscored the large variation in time-to-peak blood buffering capacity (e.g., highest recorded [${\text{HCO}}_{3}^{-}$]) between the participants (range from 30 to 180 min) (7), despite a per kilogram body mass dosing regimen and 24-h dietary replication protocols. Subsequently, these data have been followed by a series of studies assessing the intra-individual reproducibility of blood buffering profiles (14, 16, 18) and exercise performance under varying doses of NaHCO_3_ (14, 16). Collectively, these studies provide preliminary evidence suggesting that adjusting the start of a competitive effort to commensurate with an individual's peak blood buffering response at rest may result in better outcomes in terms of GI distress and exercise performance (14, 16, 19).

Although promising, the importance of timing performance trials to coincide with an individual's peak blood buffering capacity has not yet been investigated in world-class level athletes. For example, neither of the aforementioned studies investigating reproducibility and performance (14, 16) introduced NaHCO_3_ as it would be to a competitive athlete (e.g., in a fed rather than fasted state, in capsule rather than liquid-based solution and under the pressure of competitive situations). Moreover, neither of these studies have investigated the effect of performance timing. Miller et al. compared an individualized NaHCO_3_ ingestion protocol to a placebo and control trial in recreationally active individuals (16), whilst Gough et al. investigated the reproducibility of cycling time-trial performance under varying (but all individualized) dosages of NaHCO_3_ (14). We therefore designed this proof-of-concept study to specifically address the question of whether or not ingestion *timing* influences time-trial performance [2,000-m rowing time-trial (TT)] in elite-level rowers adhering to their own individualized pre-race strategies (e.g., nutrition, warm-up, etc.). Incorporating the 2,000-m rowing TT under these conditions of high ecological validity also provided the opportunity to further explore the complex, inter-related issues surrounding NaHCO_3_ ingestion, GI-distress, and acid-base balance; all concerns raised by previous investigations in this field (9, 11, 20). We hypothesized that 2,000-m rowing times would be improved when the TT commenced at an individual's peak blood buffering capacity as compared to a start time corresponding with the minimum IOC recommendations (60 min) (1).

## Materials and Methods

A multi-center approach was utilized to maximize the number of international competitive rowers involved. Twenty three (*n* = 23) elite rowers [lightweight rowers (*n* = 4), body weight 73.6 ± 2.1 kg; open-class rowers (*n* = 19), 93.6 ± 5.8 kg; mean (M) ± standard deviation (SD)] were recruited across two research centers [Canadian Sport Institute Pacific (CSIP; Canada) and the New South Wales Institute of Sport (NSWIS; Australia)] with identical methods and protocols conducted at both institutes. Participants were male competitive rowers able to complete a 2,000-m ergometer TT at or below 6 min 20 s [Participant 2,000-m ergometer TT personal bests (PB) ranged from 5 min 39 s (open-weight) to 6 min 14 s (light-weight) and included 13 Olympic/World-Champs team members as well as one rowing ergometer world record holder]. Fifteen of the 23 athletes all had previous experience ingesting NaHCO_3_ in various contexts, while the remaining eight (U23) were only aware of the potential benefits. All participants were informed verbally and in writing as to the nature and risks associated with the study, submitted to health screening and gave their written informed consent. All procedures in this study were approved by the respective ethics committees (Australian Institute of Sport Ethics Committee; approval code 20171205 and the University of Victoria Human Research Ethics Board; approval code 18-045) and were conducted in accordance with the Declaration of Helsinki.

### Pilot Study

Prior to the investigation, an ingestion protocol was implemented (see section Experimental Trials) to assess (a) the reliability of the ABL 80 Flex (Radiometer, Copenhagen, DK); (b) week-to-week repeatability of pH and standard (s) ${\text{HCO}}_{3}^{-}$ after a standardized ingestion of 0.3 g·kg^−1^ NaHCO_3_ in both study locations (inter and intra-reliability); and (c) to define objective parameters for identifying an athlete's individual time-to-peak blood buffering capacity [highest recorded (${\text{sHCO}}_{3}^{-}$)] after NaHCO_3_ ingestion [Note: ${\text{sHCO}}_{3}^{-}$ is only used in reference to the data collected in this study, whereas the abbreviation ${\text{HCO}}_{3}^{-}$ is used in all other instances (e.g., blood bicarbonate or other published papers unless otherwise noted)]. A total of *n* = 8 volunteers (four in each institute) completed two passive ingestion trials, conducted at the same time of day, each separated by 1-week and after the same dietary replication (see section Dietary Controls).

To facilitate arterialization, each seated participant's hand was warmed (either by heated pad or warm water) 10 min prior to obtaining a baseline sample and throughout the entire profiling session. For baseline and all subsequent measures (every 10 min for a total of 150 min starting 30 min after the final NaHCO_3_ pill was ingested), whole blood was collected in duplicate from the finger-tip into a heparinized 120 μl blood gas capillary tube and immediately analyzed for acid-base status (pH and ${\text{sHCO}}_{3}^{-}$; ABL 80 Flex). After the baseline sample, participants consumed gelatin capsules providing a total of 0.3 g·kgBM^−1^ of NaHCO_3_ (1,000 mg NaHCO_3_ per capsule over a 30 min period (e.g., 1/3 ingested at 0, 15 and 30 min) with a standardized snack and 10 ml·kgBM^−1^ fluid. The snack was designed to replicate typical pre-competition practice (with maltodextrin added to fluids where necessary to standardize carbohydrate (CHO) intake at 1.5 g·kgBM^−1^) and minimize potential GI discomfort (20). All NaHCO_3_ capsules were third-party batch tested by LGC Limited (Lexington KY, US; certification number 22,948) for prohibited substance contamination against the World Anti-Doping Association List.

### General Procedures

**Figure 1:**
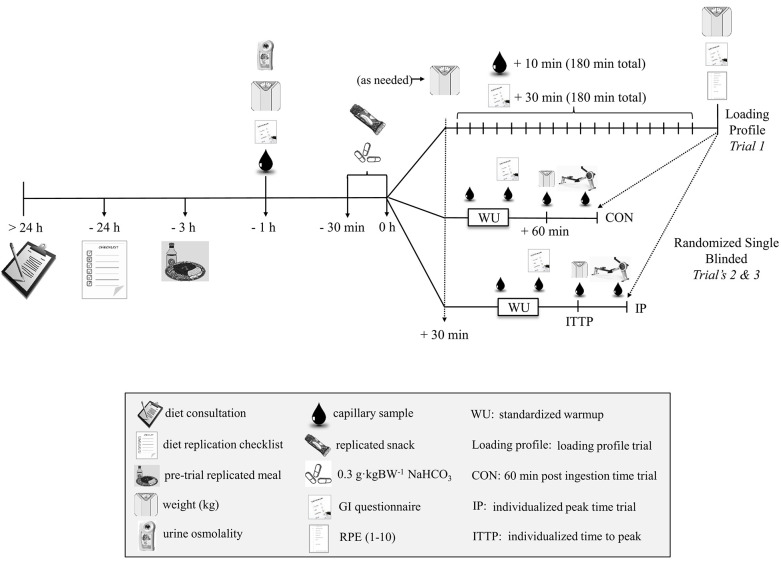
Study overview.

A study overview is provided in Figure 1. Each participant reported to the temperature controlled Exercise Physiology Laboratory's at CSIP and NSWIS on three separate occasions (loading profile followed by two randomized and athlete single-blinded deception (as outlined below) experimental trials) at the same time of day for each trial and separated by >5 and <14 days. Familiarization with this world-class cohort was established over a series of regular, 2,000-m TT efforts on the ergometer recorded during normal training and over a 12 month period prior to the study (participants had been competitive rowing training for ~ 9 ± 3 years with an average of 3 × 2,000-m TT tests/year). To implement high ecological validity, participants replicated their typical 24 h pre competition nutrition practices (macronutrient composition, volume, and timing) prior to all three trials (see section Dietary Controls).

### Individualized NaHCO_3_ Loading Profile

Participant preparation and ingestion procedures were conducted as presented previously (see section Pilot Study). At 30 min post snack, capillary sampling (in duplicate) re-commenced, where samples were obtained and analyzed every 10 min until a plateau in ${\text{sHCO}}_{3}^{-}$ was identified. The plateau was determined to have occurred when the change in three consecutive measurements were smaller than the ABL 80 technical error of measurement (TEM) (0.4 mmol·L^−1^; see section Results: Pilot Study). Sampling continued until two consecutive measurements indicated a decline in [${\text{sHCO}}_{3}^{-}$] greater than the TEM associated with identifying peak blood buffering capacity (0.6 mmol·L^−1^; see section Results: Pilot Study). Time-to-peak (min) for the IP trial was thereafter defined as the final measurement time point prior to this decline. Toilet breaks and walks around the room were considered an acceptable level of activity and GI discomfort was documented upon arrival to the laboratory and throughout this sampling period (see section Gastrointestinal Profile). Body weight was measured upon arrival to the laboratory, before and after all toilet visits and finally at the end of sampling (Avery Berkel, Model HL120, Smethwick, UK). Total fluid volume over the loading period was also recorded, along with urine specific gravity (USG; Atago PAL-10S, Tokyo, JP) at the start of the trial to ensure similar biological states between trials. From the blood acid-base data collected over the 180 min time period, peak [${\text{sHCO}}_{3}^{-}$] and time-to-peak after NaHCO_3_ ingestion was identified for the purpose of timing the start of the two subsequent experimental trials.

### Experimental Trials

After completing the individualized NaHCO_3_ loading profile, two experimental exercise performance trials were randomly administered under the following timing conditions:

- Minimum IOC consensus timing recommendation (CON): 2,000-m rowing TT performed 60 min post ingestion of 0.3 g·kgBM^−1^ NaHCO_3_.
- Individualized Timing Peak (IP): 2,000-m rowing TT performed at the participant's individual peak [${\text{sHCO}}_{3}^{-}$] (determined from the profiling) after ingesting 0.3 g·kgBM^−1^ NaHCO_3_.

As the primary aim of this study was to test whether or not it is critical to commence exercise performance trials at an individual's peak blood buffering capacity, the CON trial needed to start within a time-frame that was sufficiently supported by evidence (2–5). Therefore, when considering the available evidence suggesting individualized NaHCO_3_ supplementation might extend out the peak buffering capacity timeframe for most athletes (8, 14, 16, 18), we assert that investigating the earliest time supported by IOC guidelines (60 min) (1) compared to individual athlete time-to-peak had the potential to provide the best proof-of-concept scenario to test this effect (particularly given the logistical restraints of working with this elite population).

For the two experimental trials, participants were asked to replicate their training for 48 h before each TT and to also replicate their 24 h dietary intake (see section Dietary Controls) prior to the initial NaHCO_3_ loading profile trial. For both experimental conditions, a dose of 0.3 g·kgBM^−1^ of NaHCO_3_ was administered orally in gelatin capsules following the same ingestion protocol identified previously (see section Individualized NaHCO_3_ Loading Profile). Capillary blood was sampled for acid-base balance [(pH and ${\text{sHCO}}_{3}^{-}$), see section Pilot Study] and blood lactate (BLa) (The Edge; Woodley Equipment Company, Bolton, UK) pre-ingestion (Base) (with the exception of BLa), pre-warm-up (Pre-WU), 1 min post lactate push (Post-Push), pre time trial (Pre-TT) and 1 min post completion of the TT (Post-TT). Rating of Perceived Exertion (1–10 Borg scale; RPE) was obtained at the end of each TT. Gastrointestinal symptoms were again documented throughout each experimental trial. Body weight was assessed pre-capsule ingestion and again pre-TT to monitor any fluid changes induced by NaHCO_3_.

The 2,000-m rowing TT was performed on Concept2 rowing ergometers with the display screen blinded to give only distance completed feedback (Model D Concept2, Inc., Morrisville, Vermont, US) after participants replicated their usual, pre-competition warm-up in the laboratory. Individual pre-TT warmup (reviewed by a sports physiologist) occurred under the following guidelines: category 6 (C6; lowest intensity) “erging” with a lactate priming effort of 1 min at 2,000-m race pace completed 20 min before the start of the TT [shown to improve high-intensity TT's (21)]. Participants were able to stay warm throughout the post lactate push period with C6 or lower intensity erging, interspersed with periodic power strokes (PS). No more than 3 to 5 PS/set, with no more than 2 to 3 sets and a minimum of 5 min between sets, was permitted. For the 2,000-m TT, participants were asked to perform the 2,000-m row on their own with the only feedback being distance completed. No verbal encouragement was provided.

In this world-class cohort, all of the rowers had prior knowledge of, or experience using NaHCO_3_ and were aware of the contemporary approaches (e.g., individualized timing protocols) being trialed in various sports. To properly mitigate any performance expectancy related to individualized supplement timing, a quadruple cross-over design would have been required. Four, 2,000-m TT's was not possible, therefore single-blinded deception around whether or not the athletes received NaHCO_3_ was used to mask the potential belief that an individualized timing protocol might benefit performance over a standard timing (22). Therefore, although participants received NaHCO_3_ prior to both 2,000-m rowing TT sessions (CON and IP), they were informed in advance of the study that they would be randomly assigned to either placebo or NaHCO_3_ for either the CON or IP trials. Participants were informed that the placebo would contain calcium carbonate, and would be designed to look, taste and produce side effects similar to the NaHCO_3_ capsules without the possible performance enhancing effects. After each trial participants completed a deception questionnaire to determine the success of the blinding (63% believed the CON trial was the placebo supplement, when in fact, participants received NaHCO_3_ 100% of the time suggesting this approach was successful).

### Dietary Controls

Prior to the individualized NaHCO_3_ loading profiling each athlete reviewed and recorded their typical 24-h pre-competition diet with an experienced Sports Dietitian to standardize dietary intake while replicating their usual pre-competition (pre-race or pre-TT) nutrition practice. Timings of meals and snacks were optimized around training, with the last substantial “pre-race” meal standardized to be completed 3 h prior to the ingestion of the NaHCO_3_ load. Participants were provided with a detailed diet checklist based on their recall and asked to replicate before each trial [verbally confirmed prior to each trial (Table 1)].

**Table 1:** Mean ± SD macronutrient profile [carbohydrate (CHO), protein, fat], fiber and total energy (TE) during the 24 h prior to the trials and the Pre-TT snack for both IT and CON.

	**CHO (g)**	**Protein (g)**	**Fat (g)**	**Fiber (g)**	**TE (kcals)**
24 h profile (not including snack)	793 ± 402	263 ± 130	222 ± 113	60 ± 28	6,125 ± 2,862
Pre-TT snack	140 ± 16	20 ± 27	10 ± 10	7 ± 2	682 ± 121

### Gastrointestinal Profile

Participant GI-symptoms were documented before and immediately following the sampling period of the NaHCO_3_ loading profile assessment, as well as pre- and post-TT in the experimental trials, using a 100 mm Visual Analog Scale (VAS) for eight different GI-symptoms (nausea, flatulence, bloating, belching, stomach-ache, bowel urgency, diarrhea and vomiting) as adapted from Pfeiffer et al. (23).

### Statistical Analysis

For the pilot study intraclass correlation analysis (two-way mixed effects model ICCs with 95% Confidence Intervals) in conjunction with the typical error of measurement (TEM) statistic was applied to assess the reliability of the ABL 80 Flex and week-to-week repeatability (two weeks where duplicate samples were averaged for each week) of pH and ${\text{sHCO}}_{3}^{-}$ (24). TEM of peak ${\text{sHCO}}_{3}^{-}$ values were also determined using the change scores from baseline to peak value for each sampling period during the 2 weeks. Finally, time-to-peak ${\text{sHCO}}_{3}^{-}$ was recorded and used in conjunction with the TEM of peak ${\text{sHCO}}_{3}^{-}$ to provide an objective framework to determine the ingestion timing sequence for the IP trial.

For the experimental trials, descriptive data are presented as mean ± SD with all statistical analyses being completed using IBM SPSS Statistics version 25 (SPSS Inc., Chicago, US). Changes in blood acid-base, lactate profiles and body weight throughout the experimental trials were analyzed using a two-way ANOVA for repeated measures. In the event of a significant F ratio, *post hoc* comparisons were made using a Bonferroni correction. Mean differences and standard error (SE) between conditions as well as 95% confidence intervals (CI) were calculated when significant changes occurred over time, or when differences between conditions were observed. 2 km TT performance times (s), subjective exertion (RPE) and pre-trial USG recorded during the two experimental trials were compared using paired *t*-tests, with significant differences further evaluated for effect size using the Cohen's *d* statistic (0.2, 0.5, and 0.8 corresponding to small, medium and large effects, respectfully). Two-tailed statistical significance was accepted at *p* < 0.05.

## Results

### Pilot Study

**Figure 2:**
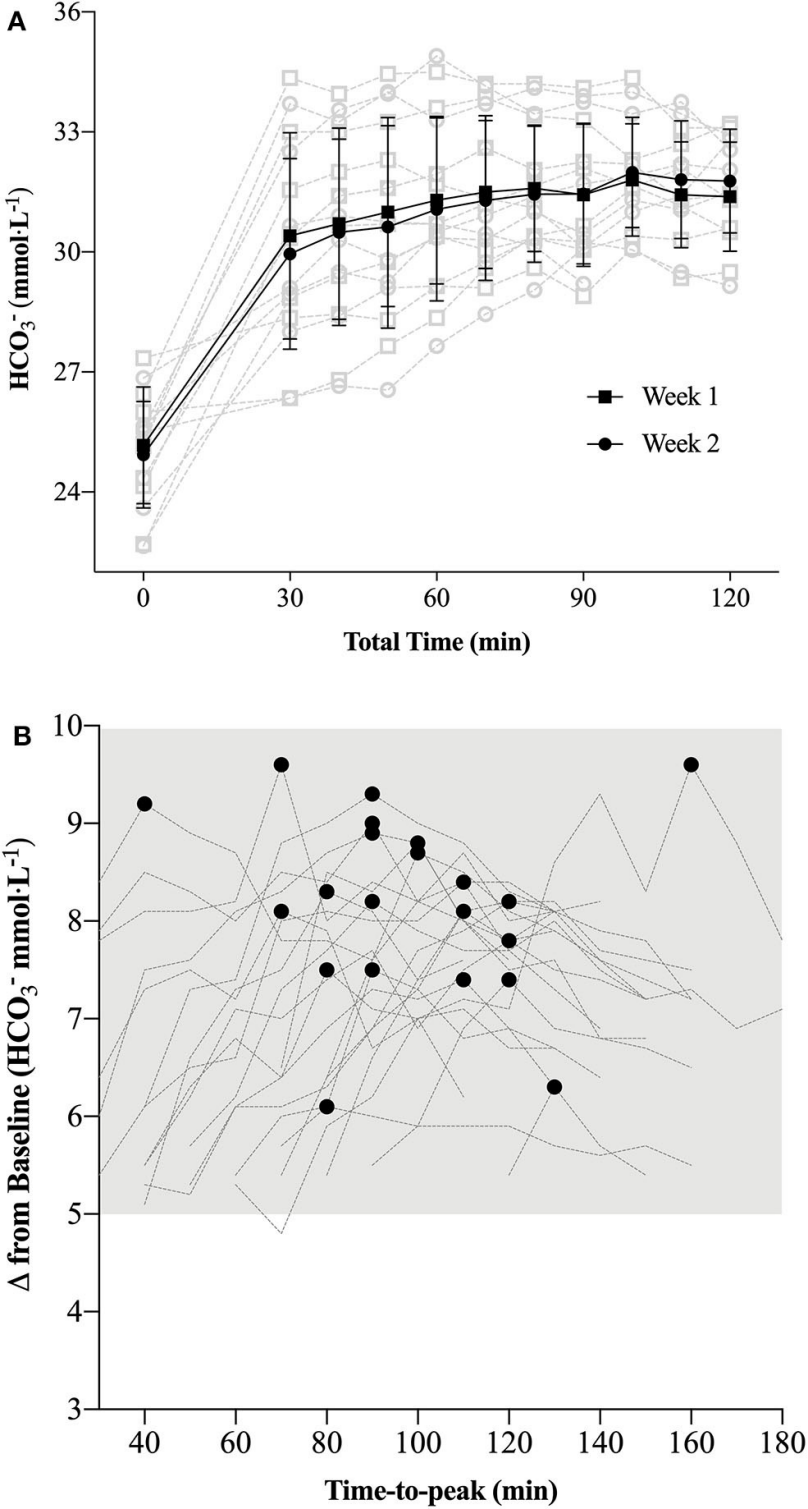
**(A)** Week-to-week standard bicarbonate concentrations ([${\text{sHCO}}_{3}^{-}$]) (mean ± SD: black; individual data: light gray) observed during the pilot study [*n* = 8; **(A)**]; **(B)**: Individual (*n* = 23) participant measurements of peak and corresponding time-to-peak as observed during the *Individualized NaHCO*_3_ *Loading Profile* trial. Each individual's time-to-peak was subsequently used to demarcate the time frame prior to commencing the time trial (TT) in the *Individualized Peak* (IP) trial.

Within-sample and week-to-week variability of the ABL 80 Flex for both pH and ${\text{sHCO}}_{3}^{-}$ is provided in Table 2and Figure 2. TEM of peak ${\text{sHCO}}_{3}^{-}$ values were 0.6 mmol·L^−1^ (95% CI 0.4 to 1.6 mmol·L^−1^), with an average variation of 14 ± 12 min (range 0 to 30 min) in time-to peak ${\text{sHCO}}_{3}^{-}$ observations.

**Table 2:** Reliability data [Intraclass Correlation Coefficients (ICC), Confidence Intervals (CI), and Typical Error of the Measurement (TEM)] collected during the *Pilot Study* for pH and ${\text{sHCO}}_{3}^{-}$ measures obtained from the ABL 80 Flex (Radiometer, Copenhagen, DK).

**Within-sample reliability**	**ICC (95% CI)**	**TEM (95% CI)**
pH (au)	0.76 (95% CI 0.66 to 0.84)	0.02 (95% CI 0.02 to 0.04)
${\text{sHCO}}_{3}^{-}$ (mmol·L^−1^)	0.99 (95% CI 0.98 to 0.99)	0.4 (95% CI 0.3 to 0.5)
**Week-to-week variability**	**ICC (95% CI)**	**TEM (95% CI)**
pH (au)	0.30 (95% CI 0.04 to 0.59)	0.04 (95% CI 0.03 to 0.05)
${\text{sHCO}}_{3}^{-}$ (mmol·L^−1^)	0.77 (95% CI 0.59 to 0.89)	1.5 (95% CI 1.2 to 1.9)

### Experimental Trials

#### Blood Parameters

**Figure 3:**
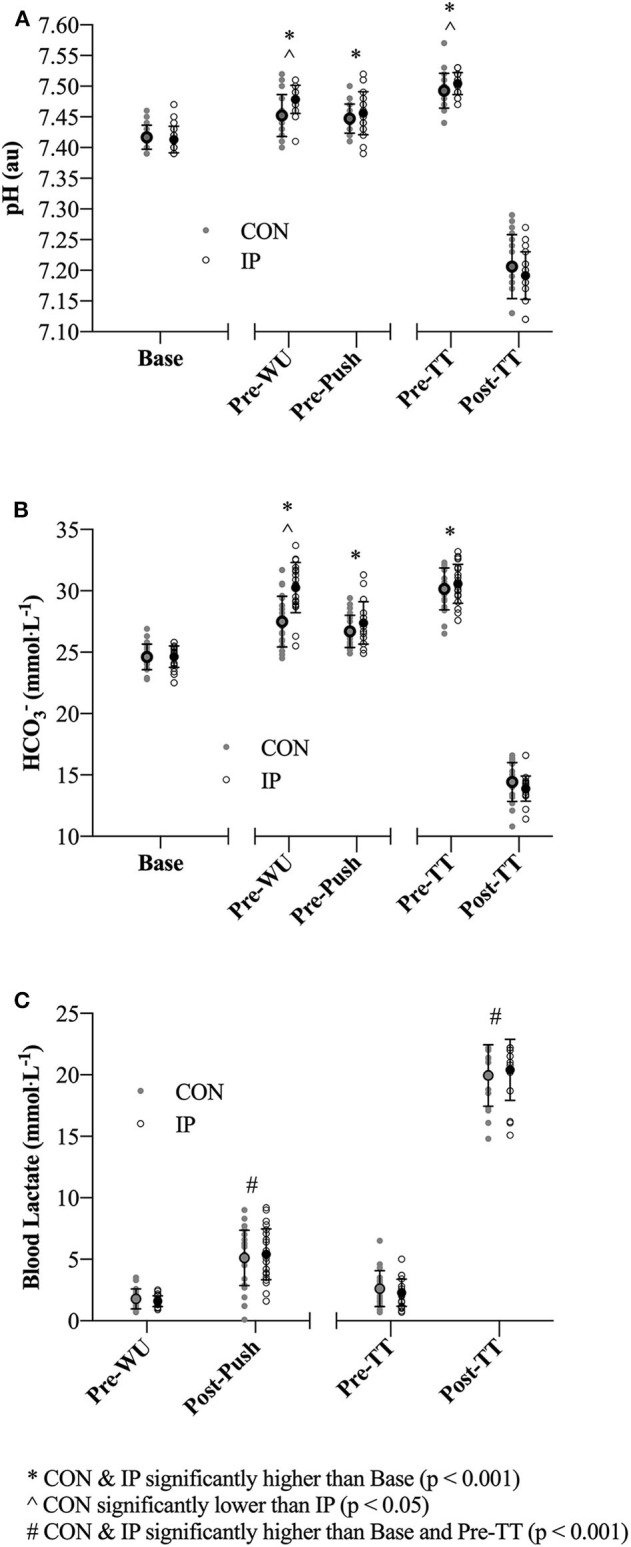
**(A–C)** Blood acid-base and lactate (BLa) measurements obtained at baseline (Base; with the exception of BLa), pre-warm-up (Pre-WU), 1 min post lactate push (Post-Push), pre time trial (Pre-TT) and 1 min post completion of the TT (Post-TT). Individual and mean ± SD data are presented for both the consensus standard (CON; gray dots) and individualized peak (IP; open black circles) trials.

Significant time effects were evident across the trial period for pH (*F* = 477.1; *p* < 0.001; η^2^ = 0.97) and ${\text{sHCO}}_{3}^{-}$ (*F* = 659.3; *p* < 0.001; η^2^ = 0.98) and consistent with induced states of metabolic alkalosis at Pre-WU and Pre-TT for both CON and IP (**Figure 3**). Significant interaction effects (pH: *F* = 4.5; *p* < 0.01; η^2^ = 0.22; ${\text{HCO}}_{3}^{-}$: *F* = 21.0; *p* < 0.001; η^2^ = 0.57) and *post hoc* comparisons revealed differences between CON and IP at Pre-WU (mean difference of 0.03 ± 0.01 au (95% CI 0.02 to 0.04 au); *p* < 0.001) and Pre-TT (mean difference of 0.02 ± 0.01 au (95% CI 0.003 to 0.03 au); *p* = 0.02) for pH, but only at Pre-WU for ${\text{sHCO}}_{3}^{-}$ (mean difference of 2.9 ± 0.4 mmol·L^−1^ (95% CI 2.0 to 3.8 mmol·L^−1^); *p* = 0.02; **Figures 3A,B**). Only a main effect of time was evident in BLa (*F* = 44.4; *p* < 0.001; η^2^ = 0.75), with Pre-WU lower than Post-Push (mean difference of 3.6 ± 0.5 mmol·L^−1^ (95% CI 5.0 to 2.1 mmol·L^−1^); *p* < 0.001) and Pre-TT lower than Post-TT (mean difference of 15.4 ± 2.2 mmol·L^−1^ (95% CI 22.2 to 8.6 mmol·L^−1^); *p* < 0.001) being significantly higher than Pre-WU and Pre-TT (**Figure 3C**).

#### 2,000-m TT Performance

**Figure 4:**
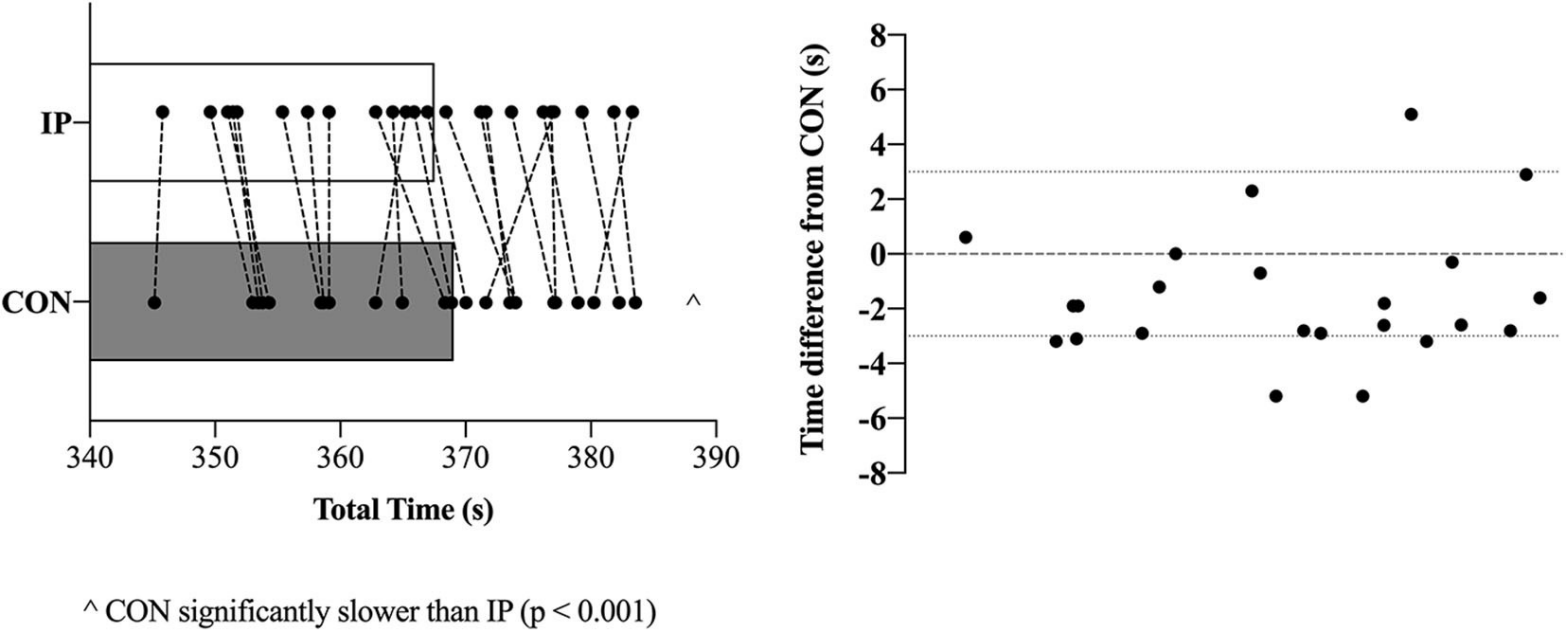
Two thousand-metres rowing time trial (TT) performance times in seconds (individual and mean ± SD data) for both the consensus standard (CON; gray bar) and individualized peak (IP; open black border) trials (left). Individual time differences (+ or – in (s) from CON) are presented in the figure on the right.

Performance times were significantly different between CON (369.0 ± 10.3 s) and IP (367.0 ± 10.5 s) [mean difference 1.5 ± 2.4 s (95% CI 0.5 to 2.6 s); *p* = 0.007; *d* = 0.15; **Figure 4**]. Of the 23 rowers, 18 improved their times in the IP trial with 11 participants at or above a 3 s improvement (**Figure 4**). There were no differences in ratings of perceived exertion scores between CON (8.9 ± 1.1) and IP (9.2 ± 0.9) after completing the TT (*p* = 0.42).

#### USG, Body Weight and GI Symptoms

**Figure 5:**
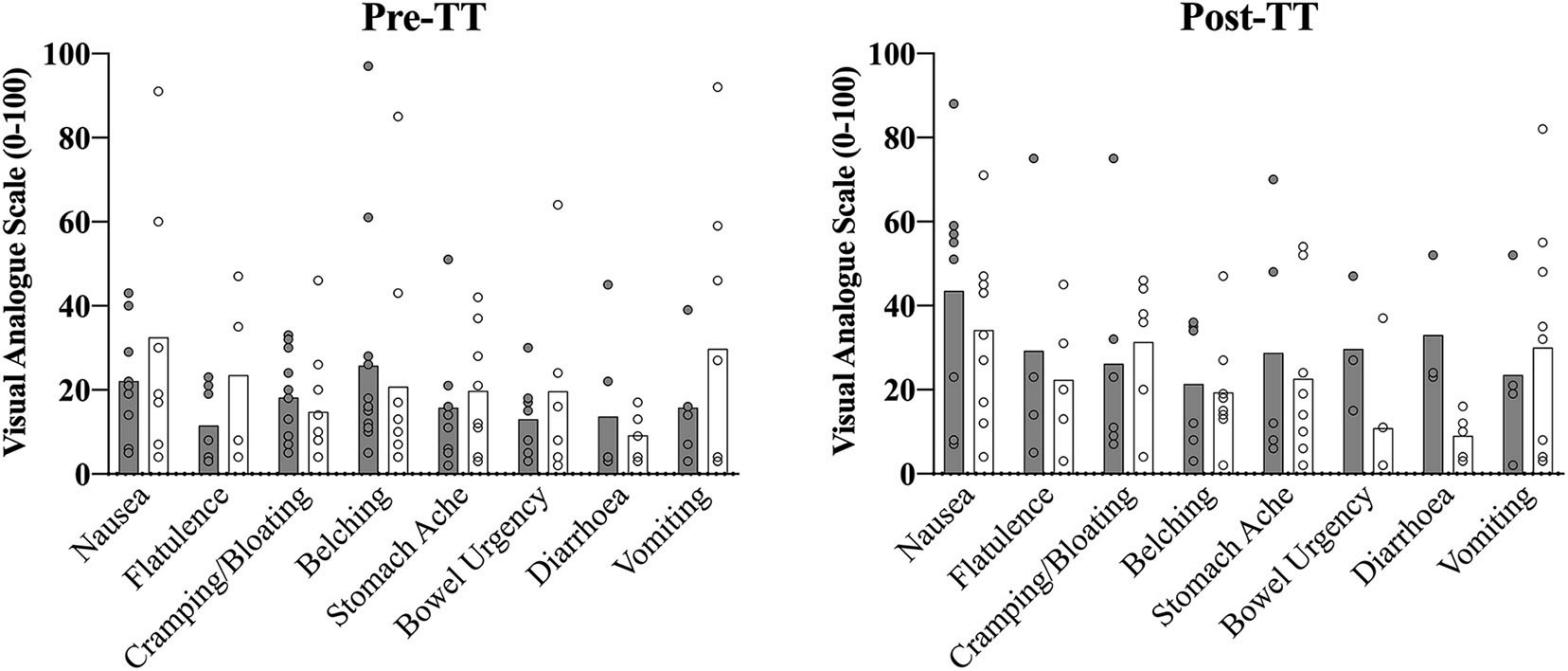
Summed descriptive (individual and mean ± SD) symptom data collated from the GI questionnaire (0–100 point visual analog scale) prior to the start of the 2,000-m TT (Pre-TT) and upon completion (Post-TT) for both the consensus standard (CON; gray bar) and individualized peak (IP; open black border) trials.

USG was not different prior to the two experimental trials (CON: 1.013 ± 0.008; IP: 1.015 ± 0.008; *p* = 0.20). Significant time effects were evident across the trial period for BM (*F* = 5.6; *p* < 0.01; η^2^ = 0.24), with a mean increase in weight of 0.35 ± 0.13 kg (95% CI 0.10 to 0.68 kg; *p* = 0.04) from Base to Pre-WU. Post-TT weights had returned to Base levels and were not different (Base: 89.77 ± 2.3 kg; Post-TT: 89.84 ± 2.2 kg; *p* = 1.0). No differences were evident between conditions (*p* = 0.33). For descriptive purposes only, GI symptoms are presented (mean ± SD) for each symptom at Pre-TT and Post-TT during the CON and IP trials (**Figure 5**).

## Discussion

A growing body of evidence suggests a number of factors may affect the efficacy of the supplement NaHCO_3_ as a strategy to mitigate fatigue in the context of exercise performance (7, 8, 14, 16–18). These factors are primarily related to the complex interplay between ingestion strategies as they relate to changes in peak blood buffering capacity (e.g., dose-response), GI distress and ingestion timing. The aim of the present study was to specifically address the issue of ingestion *timing* by examining whether adjusting start times to coincide with an individual's peak blood buffering capacity after NaHCO_3_ supplementation would influence 2,000-m rowing TT performance. Our approach of individualizing the time-to-peak was successful as Pre-WU [${\text{sHCO}}_{3}^{-}$] was nearly 3 mmol·L^−1^ greater (*p* = 0.02) for IP than CON (**Figure 3B**), although this difference diminished in the experimental trials after the addition of the warm-up (Pre-TT ${\text{sHCO}}_{3}^{-}$ values: CON ~ + 5.5 mmol·L^−1^; IP ~ + 6 mmol·L^−1^). The present study also demonstrated a small but significant performance effect of an individualized NaHCO_3_ ingestion strategy [IP (367.0 ± 10.5 s) vs. CON (369.0 ± 10.3 s); *p* = 0.007; *d* = 0.15]. Moreover, 18 of the 23 participants improved their times in the IP trial, with 11 participants at or above a 3 s improvement (**Figure 4**). Given the caliber of the athletes in this study, these findings provide preliminary support for individualizing ingestion timing strategies.

A number of recent independent investigations have clearly demonstrated the high degree of inter-individual variability associated with time-to-peak buffering capacity after NaHCO_3_ ingestion, reporting peak ${\text{HCO}}_{3}^{-}$ concentrations ranging between 10 and 150 min post ingestion after a similar dose of 0.3 g·kgBM^−1^ (7, 18, 25). Indeed, the inter-individual variability has also been cited as a possible contributing factor toward inconsistencies of erogenicity observed in some studies (8, 17), and highlighted in contemporary reviews as an area for further study (6, 26). With specific reference to rowing, it is plausible that this inter-individual variability may have influenced the performance outcomes of many of the studies observing minimal to no effect of NaHCO_3_ supplementation (9–12, 27). Although we observed less than a two second (~ 0.5%) difference in performance times (**Figure 3**), in practical terms this would equate to greater than a boat's length in competition. Even in this world-class cohort where we consistently observe 2,000-m CV's of 0.5 to 1.4% (28), the small but positive effect (*d* = 0.15) may be worth considering when developing a supplementation strategy for the elite competitor, particularly in light of the prevalence of small effects identified in this cohort (5). Though our findings tentatively support the concept of individualizing timing strategies with this supplement, further research is required to determine whether performance outcomes after individualization is due to differences in absolute changes blood [${\text{HCO}}_{3}^{-}$] or some other mechanism (14, 16, 18).

Although speculative, as we only investigated the effect of timing, the results of the present study may also suggest that the absolute change in blood buffering capacity (**Figures 3A,B**), rather than timing exercise commencement to coincide at an individual's peak blood ${\text{HCO}}_{3}^{-}$ concentration, may be more relevant when considering NaHCO_3_ as a supplement. As demonstrated in a recent review (6), the ergogenic potential of consuming 0.3 g·kgBM^−1^ NaHCO_3_ appears to improve substantially when the concentration of blood ${\text{HCO}}_{3}^{-}$ is >5 to 6 mmol·L^−1^ above typical values (found in 17 of 19 studies reviewed). In the present study both CON (~ + 5.5 mmol·L^−1^) and IP (~ + 6 mmol·L^−1^) ${\text{sHCO}}_{3}^{-}$ concentrations were elevated above 5 mmol·L^−1^ prior to the start of each of the respective time trials. Furthermore, during the *NaHCO*_3_ *Loading Profile* the time duration post-supplementation that participants were > 5 mmol·L^−1^ was ~ 100 ± 20 min (range 40 to 160 min; Figure 2B), respectively; suggesting the potential for a buffering performance window. Though the theoretical premise of a minimal buffering threshold (e.g., 5 to 6 mmol·L^−1^) or buffering window has merit, further research is required to directly test these hypotheses within the context of “real-world” performance parameters.

In support of the previous statement, the similar elevation in pre-TT [${\text{sHCO}}_{3}^{-}$] observed in both performance trials (Figure 3B) also clearly demonstrates the effect of a typical high-intensity warm-up on blood acid-base kinetics, as only one participant achieved peak blood buffering capacity at the 60 min post-ingestion time point during the passive profiling trial (Figure 2B). This finding has practical significance, as the warm-up in this study was constructed by each individual athlete (within prescribed guidelines) and mimicked their own “pre-race” warm-up strategy. Presumably, either the proportion of high-intensity efforts or warm-up length facilitated an increased rate of ${\text{HCO}}_{3}^{-}$ appearance in the blood as compared to a purely passive ingestion environment, essentially equating the buffering capacity between the two conditions. Irrespective of the similar pre-TT [${\text{sHCO}}_{3}^{-}$], we ultimately cannot dismiss the performance improvement observed in the IP trial (Figure 4). Given the similar GI responses (Figure 5) and blood buffering concentrations between trials, we cannot speculate as to causation in this regard. In practice, however, it may be unnecessary to undertake the costly and time-consuming exercise of identifying an individual athlete's peak blood buffering capacity, when measured baseline and a “one-off” measure just prior to exercise will ensure ${\text{HCO}}_{3}^{-}$ concentrations are significantly elevated (e.g., > 5 mmol·L^−1^) post supplementation.

Presently, there are no universally accepted methods for determining peak blood buffering capacity after NaHCO_3_ ingestion. Miller et al. used a single visit where peak [${\text{HCO}}_{3}^{-}$] and pH values were visually determined during 160 min of sampling (16). Similar to our pilot study results (Figure 2A), after demonstrating a high degree of reproducibility in time-to-peak [${\text{HCO}}_{3}^{-}$] (18), Gough et al. also used a single visit to determine time-to-peak ${\text{HCO}}_{3}^{-}$ over a 180 min time frame post 0.2 and 0.3 g·kgBM^−1^ NaHCO_3_ ingestion (14). Commendably, these authors reported individual participant data to complement the mean ± SD absolute ${\text{HCO}}_{3}^{-}$ change (mmol·L^−1^) scores across the resting and two experimental trials (4 km cycling TT). Although mean differences in peak values were between ~ 0.8 and 0.9 mmol·L^−1^, the range of difference scores across the three 0.3 g·kgBM^−1^ NaHCO_3_ trials was over 3 mmol·L^−1^ within individuals at the high end (14), suggesting a large degree of intra-individual variability. The present study, using a combination of time-to-peak and TEM from the change scores (baseline to peak) over the 2 week pilot study (to objectively determine peak ${\text{sHCO}}_{3}^{-}$) still demonstrated a relatively large amount of variability across peak ${\text{sHCO}}_{3}^{-}$ values (0.6 mmol·L^−1^; 95% CI 0.4 to 1.6 mmol·L^−1^). Given the inter- (7, 8, 17) and intra-individual variability associated with identifying time-to-peak ${\text{HCO}}_{3}^{-}$ values, our small but significant difference in performance times between trials, and assuming the athlete will replicate all dietary practices prior to competition, we recommend identifying the time course where an absolute increase of > 5 mmol·L^−1^ (${\text{HCO}}_{3}^{-}$) occurs after ingesting 0.3 g·kgBM^−1^ NaHCO_3_ as a criterion standard (2).

Despite implementing rigid 24 h dietary controls, a pre-race standardized ingestion strategy (10 ml·kgBM^−1^ fluid and 1.5 g·kgBM^−1^ CHO) based on previous best practice recommendations (20) and athletes' preferred pre-competition diet, all athletes in the present study experienced at least some degree of GI distress, although the majority of it was minor (Figure 5). Regardless of severity, athletes will be reticent to use this supplement should it cause distraction during competition. Indeed, four of the top performing rowers in this study have not used NaHCO_3_ during competition for this reason. Empirically, Saunders et al. have demonstrated improved exercise capacity when individuals do not experience GI discomfort after NaHCO_3_ supplementation, albeit in recreationally trained participants (8). Given our participant cohort, we cannot speculate further as to whether the GI distress was directly related to the NaHCO_3_, anxiety experienced by the rowers prior to the TT, the extreme intensity of the TT itself or a combination of factors. As suggested by numerous authors (2, 5, 6), GI distress associated with NaHCO_3_ ingestion may be one of the primary factors in this supplement not reaching its ergogenic potential. Indeed, many contemporary studies have documented the commonly experienced side-effects (e.g., bloating, cramping, diarrhea, etc.) (8, 14, 18), and have even attempted to categorize these symptoms according to severity (16). However, as of yet there have been no direct causal links established between the severity of GI distress from NaHCO_3_ ingestion and a decline in exercise performance. Moreover, the large disparity in the literature between ingestion protocols (e.g., capsules, liquid, fasted vs. fed-state), timing and nutritional control render between-study symptom comparison difficult. In the present study, although GI symptoms were similar regardless of condition, any opportunity to minimize the distractions associated with GI distress is logical and thus supports, where possible, prolonging the time period between ingestion and the start of competition.

One additional note in considering the ingestion strategy of the present study was the weight gain experienced by the athletes during both loading protocols. Although the BM increase was nominal for a weight supported sport such as rowing (<0.5%) and most likely a result of the 10 ml·kgBM^−1^ fluid administered during pill ingestion, it is worth mentioning given the potential for this supplement to be used in weight dependent (e.g., triathlon team relay, middle distance running etc.) and weight category sports (mixed-martial arts, boxing, etc.). Moreover, when separated from the group, the weight gain appeared more pronounced in the lightweight (72.5 kg) rowers (~ 0.6 kg increase; *n* = 4). To our knowledge, only one study has investigated the potential fluid increases after incorporating NaHCO_3_ (ingested within the typical range) into a rehydration strategy to offset the effects of acute dehydration (12). Kupcis et al. observed a greater increase in BM during a 2 h loading sequence compared to the present study (~ 1.5 vs. 0.4 kg), albeit total fluid intake was over double the total volume (10 ml·kgBM^−1^ in the present study compared to 22 ml·kgBM^−1^). However, these authors also did not observe any impact on performance (2,000-m rowing TT) compared to a nutrition/fluid-matched placebo control (12). Ultimately, when considering whether to implement a NaHCO_3_ loading strategy it is worth noting that the expected body weight gain from NaHCO_3_ ingestion is likely to be much less deleterious in weight supported (or independent) sports (rowing, canoe-kayak) than weight dependent sports (e.g., running, road cycling).

We acknowledge the perceived limitation in this study of not having a true placebo trial. However, the primary aim of this study was not to determine the efficacy of NaHCO_3_ ingestion on 2,000-m rowing TT performance, as the potential ergogenic effect of this supplement has been shown previously across many published reviews and meta-analyses (2–5, 25, 29, 30). Rather, we sought to specifically address the question of whether or not ingestion timing impacts TT performance in an ecologically valid context (e.g., with dietary and warm-up conditions typically used by elite rowers). In an attempt to counterbalance our lack of a traditional placebo trial, deception was used to minimize the potential belief effect of timing (31). Thus, some level of expectancy was possible. When considering that coaching staff limited our time trial opportunities (i.e. two), and the fact that our time frame for carrying out the study was constrained, we agreed that the only way to mitigate the belief effect was to deceive the athletes about whether or not they were actually receiving NaHCO_3_ in each trial. As nearly 65% of the athletes believed that the CON trial was indeed a placebo trial, we are confident this strategy was effective in “blinding” the athletes to the exact supplement they received. Given the limited peer-reviewed data available on this supplement in world-class athletes, and considering the inherent constraints of conducting research in a high performance environment (e.g., coach “buy-in,” training and competition schedules only allowing for 2 × 2,000-m TT-tests), we felt conducting the study using the 60 min timing as a control for comparison against individual peak was warranted despite the somewhat non-traditional research design.

Ultimately, the findings of the present study may support targeting the onset of a competitive effort to coincide with an individual's peak blood buffering capacity window after NaHCO_3_ supplementation if working with competitive athlete cohorts. Similarities in blood buffering changes after warm-up, however, would suggest this effect was not a result of any meaningful differences in blood alkalinity. Although a number of meta-analyses have suggested that ensuring the athlete has reached a minimum of a 5 mmol·L^−1^ absolute increase in blood [${\text{HCO}}_{3}^{-}$] may also be important to maximize the effectiveness of this supplement (2, 26, 27), further study is required to directly test this threshold hypothesis. Regardless, understanding the total timeframe of this increase should allow for greater flexibility in the timing of NaHCO_3_ supplementation (32). Moreover, given the extended time-frame above this 5 mmol·L^−1^ mark observed in all athletes (Figure 2B), we would recommend future research investigate the potential of this “window of opportunity” rather than focusing solely on peak blood buffering values. The flexibility in supplement timing across a potential performance window may help with individual GI issues, pre-competition food intake timing preferences and/or sport rules dictating warm-up time, check-in time or similar logistical constraints. In terms of supplement tolerance, even when incorporating a “tried and true” pre-race nutritional strategy that includes adequate fluid, carbohydrate and delayed ingestion timing, practitioners can expect some athletes to experience at least minor GI disturbances. Indeed, future work in this area may eventuate in eliminating this issue altogether (15, 32). Finally, those athletes participating in weight dependent sports and considering NaHCO_3_ supplementation should be conscious of acute increases in body weight associated with ingestion protocols designed to minimize GI distress.

## Data Availability Statement

The raw data supporting the conclusions of this article will be made available by the authors, without undue reservation.

## Ethics Statement

The studies involving human participants were reviewed and approved by Australian Institute of Sport Ethics Committee; approval code 20171205 and the University of Victoria Human Research Ethics Board; approval code 18-045. The patients/participants provided their written informed consent to participate in this study.

## Author Contributions

SB, TS, GS and JS conceptualized and designed all aspects of the study. All authors contributed to data collection, analyses, writing of the manuscript, and agree to be accountable for the content of the work.

## Conflict of Interest

The authors declare that the research was conducted in the absence of any commercial or financial relationships that could be construed as a potential conflict of interest.
